# Development of DNA tetrahedron-based drug delivery system

**DOI:** 10.1080/10717544.2017.1373166

**Published:** 2017-09-11

**Authors:** Yue Hu, Zhou Chen, He Zhang, Mingkai Li, Zheng Hou, Xiaoxing Luo, Xiaoyan Xue

**Affiliations:** a Department of Pharmacology, Fourth Military Medical University, Xi’an, China;; b Student Brigade, Fourth Military Medical University, Xi’an, China

**Keywords:** Nanotechnology, DNA tetrahedron, programmable structures, modifications, drug delivery system

## Abstract

Nanocarriers of drugs have attracted significant attention to tackle the problems of drug resistance or nucleic acid drug delivery, which can optimize pharmaceutical parameters and enhance the cellular uptake efficiency. Nowadays, DNA nanostructure presents an opportunity in the field of nanomaterial due to its precise control in shape and size, excellent biocompatibility, as well as multiple sites for targeting decoration. DNA tetrahedron, which is stable and easily synthesized, is used for various applications, including nuclear magnetic resonance imaging, molecular diagnosis, targeting drug delivery, and so on. In this review, we will discuss the applications of DNA tetrahedron about drug delivery, intracellular routes and its fates. Also challenges and possible solutions for developing DNA tetrahedron-based drug delivery system are detailed.

## 1. Introduction

Nanotechnology possesses a promising application in drug delivery system, considering that nanodrugs can decrease dose dependence and side effects due to its special pharmacological characteristics, such as prolonged half-life, higher bioavailability and the ability to accumulate in specific tissues (Wang et al., 2016). Although a multitude of nanocarriers, such as lipid nanoparticle, cationic polymer, and carbon tube, exist, few materials can be applied in clinic. In 1991, Chen et al. created a new era of biomaterial by presenting a programmable 3 D DNA nanotechnology (Chen & Seeman, 1991). In 2006, DNA origami emerged remarkably, utilizing long DNA chains to form a specific structure with short chains stapling them precisely (Rothemund, 2006). Since then, scientists have combined DNA structures with functional molecules for diverse applications, including biosensors, imaging *in vivo*, drug delivery system, etc. Based on the canonical Watson–Crick base pairing, DNA nanostructure, which is stabilized by strong hydrogen bond, exhibits excellent properties compared with traditional materials that are summarized as follows: (1) precise control in sizes and shapes; (2) biocompatibility and nontoxicity; (3) less susceptible to nuclease and cell lysate (Mei et al., 2011); (4) easy targeting decoration in multiple sites; and (5) smart drug delivery (Fakhoury et al., 2014). The shapes of DNA structures are numerous; tetrahedron (Goodman et al., 2005), octahedron (Andersen et al., 2008), icosahedro (Bhatia et al., 2011) as well as tube (Ren et al., 2016) are commonly studied. Tetrahedron is the most popular because of its high productivity, rigid structure, and outstanding stability (Goodman et al., 2005).

DNA tetrahedron consists of four or more single-chain DNA which can self-assemble by base pairing in a specific saline solution (Goodman et al., 2004). As a drug carrier, there exist three main approaches to connect DNA tetrahedron with drugs: (1) pre-linking the drugs, mostly nucleic acids, at the 5′ or 3′ end of single strands before self-assembling; (2) designing an overhang that does not interfere with the formation of DNA tetrahedron, and then combining the drugs through the complementary sequence with the overhang; (3) setting the drugs in the DNA double helices by physical conjugate. Although DNA nanostructure has been elaborated in many high-quality reviews, the detailed description is scarce as a kind of drug carrier. The current article focused on the application of DNA tetrahedron for delivering drugs, summarized its possible intracellular routes, and discussed the challenges for developing DNA nanostructure-based drug delivery system and some solutions to these challenges.

## 2. Applications of DNA tetrahedron in the field of drug delivery

DNA nanostructure represents an exciting new avenue for developing a drug delivery system. Considerable researches have proven that all sorts of nucleic acid drugs and some small chemical compounds could be successfully linked to DNA nanostructure and delivered into the cells, indicating the immense potential biomedical application of DNA nanostructures (Table 1).

**Table 1.** Applications and modifications of DNA tetrahedron in the field of drug delivery.

Cargo		Connective approach	Modification	Cell line *in vitro*	*In vivo*	Ref.
Small molecules	Doxorubicin	Insetting	_L_-DNA	SCC7 cells	Y	(Kim et al., 2016)
			Aptamer	MCF-7 cells	N	(Dai et al., 2016)
			Aptamer & Folic acid	HT-29 cells	N	(Sun et al., 2017)
			Tumor-penetrating peptide	U87MG cells	N	(Xia et al., 2016)
			_L_-DNA	HeLa cells	Y	(Kang et al., 2017)
	Methylene blue	Insetting	/	B16F10 cells SCC7 cells MDA-MB231 cells	Y	(Kim et al., 2016)
	Actinomycin D	Insetting	/	*Escherichia coli Staphylococcus aureus*	N	(Setyawati et al., 2014)
Nucleic acid	CpG	Pre-Linking	/	RAW264.7 cells	N	(Jiang Li et al., 2011)
	siRNA	Overhang	Folic acid	HeLa cells	Y	(Hyukjin Lee et al., 2012)
	ASOs	Loop	Lipofectamine 2000	Hela cells C2C12 cells MCF7 cells	N	(Keum et al., 2011)
		Insetting	PNA	Escherichia coli	N	(Readman et al., 2017)
	Aptamer	Overhang	/	HeLa cells NIH3T3 cells	N	(Charoenphol & Bermudez, 2014)
		Pre-Linking	_L_-DNA	HeLa cells NIH-3T3	N	(Kim et al., 2014)
		Overhang	/	MCF-7 cells A549 cells HT-29 cells	N	(Dai et al., 2016)
		Overhang	Folic acid	HT-29 cells	N	(Sun et al., 2017)

Y: yes; N: no.

### 2.1. Small molecules

As a promising nanocarrier, DNA tetrahedron provides enough spaces for small molecules because of its cage-like structure. Doxorubicin, an anticancer drug, can effectively be inserted in GC-rich regions of DNA. Kim et al. (2013) demonstrated that combining doxorubicin and DNA tetrahedron to form DOX@Td could stimulate uptake of doxorubicin in the doxorubicin-resistant cell as much as wild types, which avoided integration with P-glycoprotein and recognition of efflux pump compared with free doxorubicin. The work illustrates that DNA tetrahedron is a good candidate for delivering drugs in clinic to overcome the drug resistance in cancer cells, which makes a significant difference in the progress of treating cancers. The same group constructed an _L_-DNA tetrahedron to deliver doxorubicin *in vivo*, finding decreased clearance and increased initial concentration (C0), half-life, and area under the curve (AUC). In addition, one with 17-mer per side showed higher internalization than that with 30-mer (Kang et al., 2017). They emphasizes the importance of DNA tetrahedron’ s sizes and conformations when it is used for delivering drugs *in vivo*, provoking researchers into optimizing this carrier by altering the physicochemical properties, such as charge numbers and diverse modified groups. Nowadays, Zhang et al. (2017) used DNA tetrahedron as a carrier of doxorubicin with modifying by two affibody molecules, exhibiting greater selectivity toward as well as cytotoxicity of breast cancer cells overexpressing HER2 than doxorubicin does. And the inhibition rate is two-fold at least than trastuzumab toward BT474 cells (Zhang et al., 2017). The results indicate that it is promising to modify the DNA tetrahedron with targeted molecules, but still difficult to find the most efficient system in order to be applied clinically.

Moreover, methylene blue (MB), which is used for photodynamic therapy but not stable with low-delivery efficiency, can also interact with DNA duplexes. Kim et al. loaded MB on a DNA tetrahedron (MB@Td), demonstrating that the cellular uptake of active MB enhanced with the help of DNA tetrahedron in the B16F10, SCC7, and MDA-MB231 cell lines. And it was obvious that the viability of cells treated with MB@Td decreased to 20–30%, but free MB only leaded to a minor cytotoxicity. What’s more, they claimed that the growth of SCC7 tumor could be suppressed *in vivo* substantially if the mice were exposed to the laser in the presence of the MB@Td (Kim et al., 2016). In addition, Setyawati et al. delivered Actinomycin D by DNA tetrahedron. The uptake efficiencies by *Escherichia coli* and *Staphylococcus aureus* were 70% and 80%, respectively, and sufficient bacterial cell death was achieved. After entering cells and being degraded by DNase, DNA tetrahedron disaggregated and released drugs to kill bacteria by inhibiting the synthesis of RNA (Setyawati et al., 2014). However, there still remains some confusions about the way of drug release from DNA tetrahedron as well as the location and accumulation levels of drugs in the intracellular environment, so further study are essential. To date, most researches focus on the small molecules that have high affinity with DNA. The applications will be extended broadly if strategies that efficiently use the cavity of DNA tetrahedron are discovered.

### 2.2. Nucleic acid drugs

#### 2.2.1. CpG

Unmethylated cytosine-guanine dinucleotide-containing oligodeoxynucleotide, as an agonist of Toll-like receptor 9 (TLR9), activates immune responses for use as vaccines to treat cancer, infectious diseases, and allergies (Bai et al., 2017). The obstacles in clinical application of CpG are the degradation by DNase in physiological conditions and low uptake efficiency by cells. Nevertheless, DNA nanostructure opens new avenues for the delivery of CpG.

To investigate the optimal structure for delivery, Ohtsuki et al. designed three different structures and observed that CpG tetrahedron entered into cells most efficiently, inducing the largest secretion amount of tumor necrosis factor-α compared with CpG tetrapodna and CpG tetragon (Ohtsuki et al., 2015). In 2011, Li et al. appended CpG sequences to a DNA tetrahedron in the vertex, leading to remarkable uptake efficiency by macrophage-like cells without any transfection agents, and produced high-level secretion of multiple inflammatory factors after recognition by TLR9. In this work, the immunostimulatory effect enhanced, accompanying with the increase in the valence of CpG motifs (Li et al., 2011). It is evidenced that DNA tetrahedron has the potential to tap into the utilization of CpG vaccines in the future, and the optimization should be considered to meet the requirement of less toxicity and more efficiency.

#### 2.2.2. Sequence-specific short interfering RNA

Sequence-specific short interfering RNA (siRNA) can trigger RNA interference (RNAi) to bind the reverse mRNA and halt the synthesis of related proteins. However, siRNA with negative charge is unstable and does not penetrate the cell member easily. In addition to chemical modifications, proper delivery material is required to transport siRNA to the target sites. DNA nanostructure is nontoxic and induces few immune responses when used to shield siRNA in comparison with conventional materials, such as liposomes and cell-penetrating peptides.

Lee et al. used DNA tetrahedron modified with folic acids to deliver 2′-OMe siRNA to cells and silenced over 50% target genes. Interestingly, they claimed that the delivery of siRNA is related to the density and spatial orientation of folic acids, which was impossible to evaluate by traditional nanoparticles. In addition, siRNA delivered by modified DNA tetrahedron accumulated in tumor and kidney, resulting in protein decrease. In addition, the blood half-life data indicated that siRNA ligated with DNA tetrahedron possesses a longer circulation time (*t*_1/2_ = 24.2 min) compared with the natural siRNA (Lee et al., 2012).

#### 2.2.3. Single-stranded antisense oligonucleotides

The application of single-stranded antisense oligonucleotides (ASOs) started in 1978 with 13-mer nucleic acids in which phosphodiester linkages are vulnerable to nucleases (Stephenson & Zamecnik, 1978). Many chemical modifications improve the ASOs stability and property, such as 2′-hydroxyl (OH), 2′-fluoro (F), 2′-hydroxymethyl (O-Me), 2′-methoxyethyl (MOE). Moreover, phosphorothioate, morpholino, and peptide nucleic acid (PNA) backbones have been milestones for the clinical utilization of ASOs. However, the modifications raise potential safety concerns; hence, protection by natural DNA can address this problem to some extent. Keum et al. designed a DAN tetrahedron with an ssDNA antisense loop to degrade mRNA and inhibit protein expression *in vitro*, with two-fold enhanced cellular uptake efficiency compared with partial duplex DNA and approximately 40% reduction of corresponding proteins (Keum et al., 2011). In view of most common structures for DNA tetrahedron to detect the mRNA in cytoplasm, the loop is likely to be the desired form to silence genes. Readman et al. incorporated targeted anti-blaCTX-M-group 1 antisense PNA (PNA4) in DNA tetrahedron. The minimum inhibitory concentration (to CTX) of an *E. coli* carrying blaCTX-M-3 was reduced from 35 mg/L to 16 mg/L in the presence of PNA4 carried by the DNA tetrahedron vector (Readman et al., 2017). Although the concentration of DNA tetrahedron is up to 40 μM which is obviously less efficient than it in eukaryocyte does, it is no doubt that there remains great room of it to be applied in the field of antisense antibacteria.

#### 2.2.4. Aptamers

Aptamers are synthetic single-stranded nucleic acids that may bind to small molecules, proteins, and other targets with high affinity and specificity. Compared with antibodies, aptamers are chemically stable, economical, and producible on a large scale. Therefore, the combination between DNA tetrahedron and aptamers has established a foundation for targeted delivery and therapy. Through overhangs formed by complementary pairs, aptamers can more easily bind with DNA tetrahedron compared with other materials.

Charoenphol et al. incorporated AS1411 aptamers into DNA tetrahedron, highlighting the increased uptake and selective inhibitions of cancer cell growth (Charoenphol & Bermudez, 2014). Meanwhile, Kim et al. assembled AS1411 and _L_-DNA tetrahedron, observing higher uptake efficiency and cytotoxicity in cancer cells than _D_-DNA (Kim et al., 2014). In Yang’s work, DNA tetrahedron delivered doxorubicin into Mucin 1-positive breast cancer cells with the help of MUC1 aptamer (Dai et al., 2016). Furthermore, modified by SL2B aptamer and folic acid simultaneously, DNA tetrahedron exhibited anticancer effects with lower concentration of doxorubicin (Sun et al., 2017). In conclusion, aptamers expand the application of DNA pyramid, and the system can be a promising strategy for targeted anti-cancer therapies.

As a novel carrier for drug delivery, DNA tetrahedron possesses significant advantages over others when used for transporting nucleic acids. On the one hand, combining drugs by precise rules of base pairing is convenient, rather than costly covalent linkage or unmanageable electrostatic forces. On the other hand, studying the spatial influence of ligands by designing sticky overhangs in various sites to combine functional molecules is possible. However, the application of DNA tetrahedron to deliver small molecules is limited to those possessing high affinity with DNA double strands. Therefore, addressing this problem to achieve further applications is possible.

## 3. Intracellular routes of DNA tetrahedron

Different from ions and amino acids, macromolecules depend on specific vesicles to enter cells by phagocytosis or pinocytosis. Phagocytosis is applied to limited cells, such as macrophages, monocytes, dendritic cells, as well as neutrophils, while pinocytosis is common in all kinds of cells, consisting of four types of mechanisms: macropinocytosis, clathrin-mediated endocytosis, caveolin-mediated endocytosis, and clathrin- and caveolin-independent endocytosis (Lee et al., 2016).

### 3.1. Phagocytosis

Phagocytosis is involved in the development and remodeling of tissue as well as immune and inflammatory response, defending foreign matters such as bacteria, viruses, and drugs (Aderem & Underhill, 1999). After recognizing the complexity of invaders and opsonin, macrophages activate actin proteins to form phagosome, which fuses with acid lysosome during its mature status, aiming to degrade external materials. The fact is that achieving therapeutic goals for particular carriers at some time is an indispensable process. For example, polymers need disassembling to release drugs, the same with pH-sensitive bonds. The rate of phagocytosis is influenced by the foreign body’s apparent parameters. Furthermore, the minimum standard of particle size is 500 nm for phagocytosis, with lower efficiency when less than 250 nm (Vonarbourg et al., 2006). The reason for this phenomenon is that opsonin binding is related to the size of the intruders. In view of the small DNA tetrahedron size (6–10 nm), phagocytosis by macrophages can be avoided to decrease inflammatory reactions and enhance efficiency to target tissues.

### *3.2. Pinocytosis pathway*s

#### 3.2.1. Macropinocytosis

It is a process driven by actin that begins with the invagination of cell membrane and formation of macropinosomes. Lipids and pertinent regulation proteins prompt macropinosomes to mature by modification (Kerr & Teasdale, 2009). Cell-penetrating proteins with abundant cationic amino acid prefer entering cells by this method, and alkaline residues is necessary in this process (Strømhaug et al., 1997). Despite the lack of selection, macropinocytosis seem to be irreplaceable in the uptake of drug carriers. Walsh et al. reported the first observation on mammalian cell delivery of DNA tetrahedron without a transfection agent, meanwhile it was evidenced that DNA tetrahedron with the aid of Lipofection exhibited the highest transfection efficiency compared with a single-stranded oligonucleotide, a partially double-stranded complex of this oligonucleotide, and a fully double-stranded duplex. Furthermore, they noticed transfected DNA tetrahedron located around the microtubule-organizing centers after a gradual movement throughout the cytoplasm, so they speculate cells internalize DNA tetrahedron by a microtubule-mediated endocytic pathway (Walsh et al., 2011). These results indicate that DNA tetrahedron has significant potential to deliver cargoes and transfection mechanisms require further exploration. In other researches, DNA tetrahedron was also internalized by DLD-1, and SW480 cells through this method (Tay et al., 2015).

#### 3.2.2. Clathrin-mediated endocytosis

Clathrin-mediated endocytosis (CME) is divided into two types, receptor dependence and receptor independence. Modified carriers with corresponding ligands can be internalized by receptors (transferrin, epidermal growth factor, etc.), which guide the target delivery in most cases. The whole course occurs in the clathrin-rich member regions, beginning with the formation of a 120-nm early endosome followed by fusing with lysosome. Furthermore, lipid nanoparticles with negative charges through this mechanism are observed. Nevertheless, depending on nonspecific electrostatic force, receptor-independence has lower uptake rates (Strømhaug et al., 1997). Although there exists no sufficient evidence that natural DNA tetrahedron enters cell through this route but DNA nanoribbon and polymeric nanocarrier do, Kim et al. (2014) found that _L_-DNA tetrahedron entered cell not only non-clathrin-mediated but also clathrin-mediated endocytosis. The results are consistent with higher uptake efficiency of _L_-DNA (Kim et al., 2014).

#### 3.2.3. Caveolin-mediated endocytosis

Caveolins are ∼20 kDa scaffolding proteins that assemble as oligomeric complexes in lipid raft domains to form caveolae, flask-shaped plasma membrane invaginations with an average size of 60 nm (Busija et al., 2017). Caveolae are present in many lines of cells, such as endothelial cells, smooth muscle cells, and fibroblasts. The caveolar vesicle does not contain any detrimental enzymes; hence, pathogens avoid navigating through this intracellular route (Hillaireau & Couvreur, 2009). Furthermore, this is an opportunity for nanoparticles to escape from endosomes (Lee et al., 2016). Kim et al. revealed that DNA tetrahedron is internalized via macropinocytosis and caveolae-mediated endocytosis pathways firstly (Kim et al., 2013). In 2014, Fan’s group illustrated the whole fate of DNA tetrahedrons during the course of cellular entry and intracellular transport of Hela cells through single-particle tracking, laying the foundation for further application. It demonstrated the fusion process of DNA tetrahedron and cellular membrane occurs over approximately 87 s. In addition to the conclusion that cellular uptake was dependent on the energy, they also found that only methyl-b-cyclodextrin (MbCD), an inhibitor for caveolin-dependent endocytosis, could reduce the uptake. The same was true with the COS-7 cell line. After endocytosis, the DNA tetrahedrons were transported to the lysosomes through tubulins. Additionally, they functionalized DNA tetrahedrons with nuclear localization signals, which help their escape from the lysosomes and location in nucleus (Liang et al., 2014).

#### 3.2.4. Other pinocytosis pathways

How endocytic pits are built in clathrin- and caveolin-independent endocytosis still remains poorly understood. At present, clathrin and caveolin-independent endocytosis are classified by the regulating GTPase (RhoA, Arf6, or cdc42). Among these, the clathrin-independent carrier/GPI-anchored protein-enriched early endosomal compartment pathway depends on Cdc42 and Galectin-3 rather than on actin to form endosomes (Sabharanjak et al., 2002), which is different from other mechanisms. In addition, other structures are internalized within any endocytic vesicle, defined by ‘lipid rafts’ with a diameter of 40 nm to 50 nm (Lee et al., 2016). And there is a lack of evidence that DNA tetrahedron is internalized by the clathrin- and caveolin-independent endocytosis.

In theory, DNA is confronted with barriers when penetrating cell membrane due to its abundant charges. However, DNA nanostructures can enter cells without any transfection reagents. As mentioned above, the discussions of mechanisms concentrate on macropinocytosis and caveolin-mediated endocytosis. However, the variation of specific routes in the different cell lines is still controversial. Furthermore, in view of rich negative charge, some researchers presume that DNA nanostructures would interact with scavenger receptors or TLRs, leading to harmful signaling cascades when crossing the cell membrane. In conclusion, efficient methods and enough evidence that enable the demonstration of the entire process have yet to be developed.

## 4. Challenges and possible solutions for developing DNA tetrahedron-based drug delivery system

**Figure 1.**
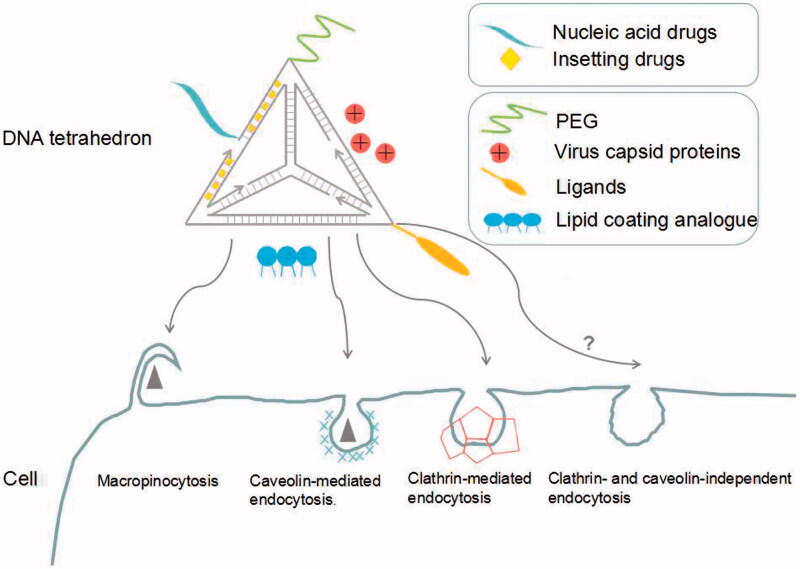
Modification strategies and intracellular routes of DNA tetrahedron in the process of delivering drugs.

As a novel drug delivery system, DNA tetrahedron faces lots of challenges such as nuclease susceptibility, low uptake efficiency, etc. However, there exist several possible solutions to optimize the system with the explorations of researchers (Figure 1).

### 4.1. Nuclease susceptibility

The achievement of DNA tetrahedron depends on its stability in both intracellular and extracellular environments. Composed of natural DNA, this carrier is vulnerable to nuclease, although more stable than linear ones because of its specific structure. Kim et al. found that utilizing _L_-DNA was more stable than natural _D_-DNA with eight-fold half-time period *in vivo*. In this work, DNA tetrahedron assembled by L-DNA delivered anticancer drugs with enhanced cellular and tissue penetration, even having a better effect than PEGylated liposomes (Kim et al., 2016). Moreover, the chemical modification of DNA in selective point is a simple method to enhance the resistance to nuclease (Dowdy, 2017). In addition to self-optimization, functionalizations with other molecules or polymers are appropriate solutions. Conway et al. (2013) modified oligonucleotide ends with hexaethylene glycol and hexanediol to increase nuclease resistance under serum conditions with lifetimes of 62 h. Inspired by natural virus, Perrault et al. enveloped DNA octahedron by PEGylated lipid bilayers against nuclease digestion with enhanced bioavailability (Perrault & Shih, 2014). In another research, Kiviaho et al. (2016) connected a brick-like DNA origami with cationic block-copolymers by the electrostatic binding, with the numbers of cationic polymers affecting the enzyme kinetics of the complexes. Furthermore, the author speculated that the polymer coating of the origami limits the accessibility of the enzymes and restricts the diffusion rate of the substrate.

### 4.2. Low-uptake efficiency

Cellular uptake is related to several factors, such as the shape, size, and charge of nanoparticles as well as the type of cells. Moreover, nanoparticles charged positively can be internalized more easily, but more vulnerable in blood circulation (Gratton et al., 2008). However, because of the negative charge, passive uptake of DNA tetrahedron can be hindered by electrostatic repulsion, which indicates that this process is energy-dependent. Given its low-uptake efficiency, various modification strategies can be adapted. The DNA nanostructure encapsulated by the viral capsid by Mikkila et al. exhibited 13-fold delivery efficiency than the naked one (Mikkila et al., 2014). His group appended a tumor-penetrating peptide (TPP) to DNA tetrahedron, increasing the uptake rate by glioblastoma cell U87MG (Xia et al., 2016). Binding to transmembrane glycoprotein neuropilin-1, TPP mediates the endocytosis of the DNA nanostructure. In some researches, Lipofectamine 2000 was used to enhance the cellular uptake efficiency (Keum et al., 2011), and exhibit dose-dependence to deliver triangular DNA structure (Wang et al., 2016). However, this strategy may lead to the changes of DNA structure’s specific property due to lipid encapsulation.

### 4.3. Escape from endosome

Endosomal escape is a critical biological obstacle in the course of delivering gene drugs, which is involved in nanoparticle types, uptake routes, and cell types (Ma, 2014). Several common mechanisms were found to stimulate the escape into cytoplasm, such as pore formation, pH-buffering effect, and fusion into lipid bilayer (Varkouhi et al., 2011). Lee et al. (2012) noticed that gene silencing activity was only observed for DNA tetrahedron with the appropriate spatial orientation of folic acid, in other words, the local density of folic acid is high. They proved that the density and location of ligands can greatly influence the intracellular trafficking pathways. Therefore, it is a feasible way to help the DNA tetrahedron escaping endosome by modifying appropriate ligands (Lee et al., 2012). To find other solutions, further compelling evidence should be presented to elaborate the fate of DNA structures after entering the cell.

### 4.4. Off-target effect

Nonspecific complementation of antisense drugs may produce inevitable side effects, so recent researches focused on target delivery to achieve precise effects. Despite the distinguished advantages, some obstacles need to be tackled, such as negative targeted gathering, lower specificity of target molecules, as well as conformation change during transporting. Lee et al. (2012) found that three folic acids are necessary at least when applying DNA tetrahedron to deliver siRNA into cancer cells, and spatial arrangements of folic acids make a difference. In the rapid development of aptamers, most target strategies of DNA structure pertain to aptamers, which have been discussed above. We can also learn from proper strategies in other delivery systems. For example, Hu et al. (2015) enclosed polymeric nanoparticles in the plasma membrane of human platelets, reducing cellular uptake by macrophage-like cells with selective adhesion to damaged vasculatures as well as pathogens. In an experimental rat model of coronary restenosis and a mouse model of systemic bacterial infection, docetaxel and vancomycin demonstrated enhanced therapeutic efficacy following this method, respectively (Hu et al., 2015). Interestingly, nanoparticles showed both characteristics when fused with erythrocyte and thrombocyte membranes.

## 5. Conclusions

DNA tetrahedron possesses a multitude of advantages over traditional carriers, such as favorable biocompatibility and biodegradability, as well as programmable structures. DNA tetrahedron conclusively has a significant potential in health science and clinical applications. However, many challenges exist as well as unexplored opportunities. First of all, structural integrity is the most noteworthy throughout its transfer to its destination. Compared with enzymatic degradation, researchers found that the low ionic concentration in cytoplasm and serum leads to easier disassembly. Although the encapsulation of DNA tetrahedron into lipid bilayer or virus capsid is an acceptable solution, the designed shape and the controlled site of ligands cannot function properly.

In fact, to address the instability and unsatisfactory cellular uptake, surface modification is undoubtedly the best choice. However, the optimal functional modification is currently chaotic due to a mass of shapes and structure of DNA nanostructures. Compared with other complicated structures, the smaller size and relatively lower charge of DNA tetrahedron makes it a promising generic core structure.

Additionally, the high cost of DNA synthesis is another concern. One gram of scaffolded megadalton-sized DNA origamis costs approximately $100,000 (Linko et al., 2015). A reduced price means that more sophisticated DNA nanostructures may be affordable and realizable. The rapid development of the technology of DNA synthesis, amplification and purification is needed.

The ultimate goal is to establish a highly efficient but cheap drug delivery system by combining the well-designed DNA nanostructures with functional molecules to overcome multiple biological barriers. Although recent researches of DNA nanostructures are prosperous and has achieved plenty, further study is necessary to apply this material in clinics and benefit human beings.
